# The Potential of Cryptophyte Algae in Biomedical and Pharmaceutical Applications

**DOI:** 10.3389/fphar.2020.618836

**Published:** 2021-02-02

**Authors:** Maryam Abidizadegan, Elina Peltomaa, Jaanika Blomster

**Affiliations:** ^1^ Environmental Laboratory, Faculty of Biological and Environmental Sciences, University of Helsinki, Lahti, Finland; ^2^ Institute of Atmospheric and Earth System Research (INAR)/Forest Sciences, University of Helsinki, Helsinki, Finland; ^3^ Ecosystems and Environment Research Programme, Faculty of Biological and Environmental Sciences, University of Helsinki, Helsinki, Finland

**Keywords:** fatty acids, sterols, carotenoida, mycosporine-like amino acids, polysaccharides, phenolics, vitamins, cryptophytes

## Abstract

Microalgae produce a variety of bioactive components that provide benefits to human and animal health. Cryptophytes are one of the major groups of microalgae, with more than 20 genera comprised of 200 species. Recently, cryptophytes have attracted scientific attention because of their characteristics and biotechnological potential. For example, they are rich in a number of chemical compounds, such as fatty acids, carotenoids, phycobiliproteins and polysaccharides, which are mainly used for food, medicine, cosmetics and pharmaceuticals. This paper provides a review of studies that assess protective algal compounds and introduce cryptophytes as a remarkable source of bioactive components that may be usable in biomedical and pharmaceutical sciences.

## Introduction

In recent years, commercial and scientific attention has remarkably boosted the interest in natural products from aquatic organisms, especially algae ‐ both macroscopic algae and microalgae. Microalgae are broadly considered as good sources of fiber, minerals, antioxidants, vitamins, pigments, steroids, lectins, polysaccharides, proteins, polyunsaturated fatty acids and other lipids (Blunt et al., 2012; Aditya et al., 2016). These products can be commercially used in a variety of applications, for example in human and animal nutrition, in cosmetics and beauty products, and for the synthesis of antibacterial, antiviral, antimicrobial and anticancer drugs (Cardozo et al., 2017; Rizwan et al., 2018).

The conversion of light energy into chemical energy by CO_2_ fixation is ten times higher in microalgae than in terrestrial plants, making the production efficiency of microalgae outstanding.

Currently, the commercial production of microalgae has been reported roughly 5,000 tons per year of dry matter (Raja et al., 2008). Almost 110 commercial products of microalgae are found in the Asia-Pacific area (Sathasivam et al., 2019). Of the estimated 200,000–800,000 microalgal species, only about 35,000 strains are scientifically described (Cheng and Ogden, 2011), only few of which are commercially employed.

**Figure 1:**
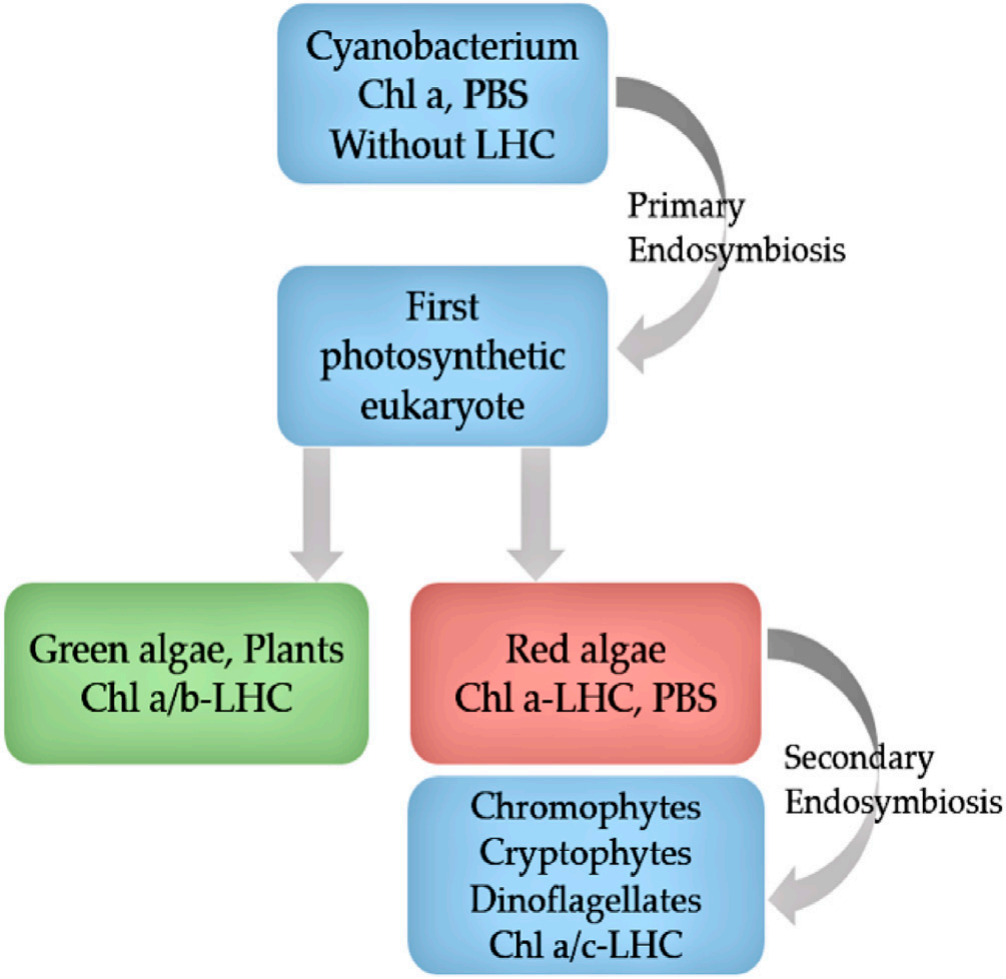
Evolution of cryptophytes according to pigment, ultrastructure and molecular phylogenic data. Chl, chlorophyll; PBS, phycobilisome; LHC, light harvesting complex (adapted from Green 2001).

**Figure 2:**
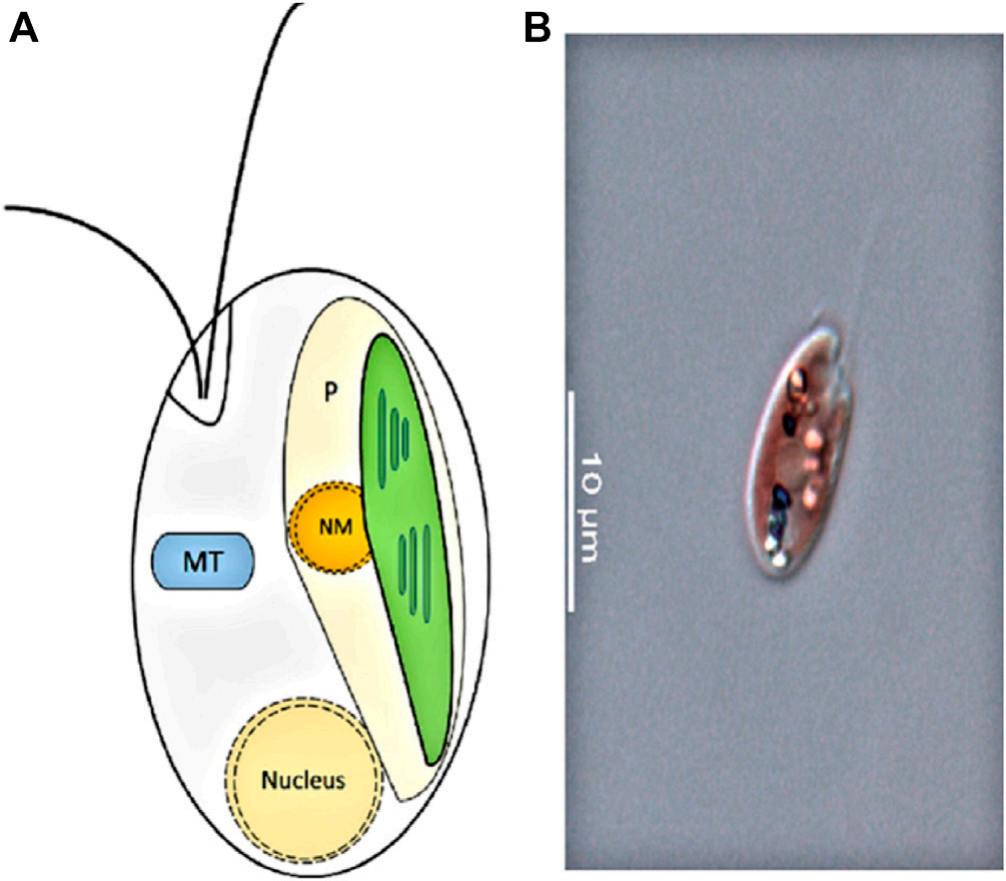
**(A)**: Cryptophyte cell structure. P, plastid; NM, nucleomorph; MT, mitochondrion (adapted from Hoef-Emden 2008). **(B)**: Photo of a cryptophyte *Rhinomonas nottbecki* n. sp. taken by Janne-Markus Rintala.

Cryptophyte algae form one of the major groups of phytoplankton, with more than 20 genera composed of 200 species (Clay et al., 2015). They are unicellular, eukaryotic algae generated from secondary endosymbiosis between a single-cell eukaryote host and a red algal predecessor (Greenwold et al., 2019) (Figure 1). The red algal ancestor has provided the cryptophyte plastid, and the ancestors’ genome forms the nucleomorph found in the plastid (Figures 2A,B). Depending on their accessory pigments, cryptophytes are bluish, reddish, brownish or green in color. Cryptophytes do not possess a cell wall, but like all chromophyte algae, they have an extra pair of membranes around their plastids. Active movements are enabled by two flagella (Figure 2). Cryptophytes are significant primary producers in both freshwater and marine habitats, and proven to be a highly important food source for secondary producers due to their exceptionally good fatty acid, sterol and amino acid profiles and concentrations that meet the needs of consumers (Brett et al., 2009; Martin-Creuzburg and Von Elert, 2009; Clay et al., 2015; Peltomaa et al., 2017). Thus far, nine cryptomonads organelle genomes have been sequenced and published, which includes three nucleomorph, one nuclear, three plastid and two mitochondrial genomes (Douglas, 1992; Kim et al., 2015).

Growth rates of most cryptophytes are considered as fairly slow (well below 0.8 div. day^−1^), and they may therefore be ignored in commercial terms. Nonetheless, in appropriate environments some strains possess higher growth rates, e.g. 1.2 div. day^−1^ (Lewitus and Caron, 1990). Due to their small cell size (below 500 µm^3^), the cell biomass of cryptophytes is low in comparison with of many diatoms and dinoflagellates, which may give an incorrect impression of the gain of effective biomass: cryptophytes lack heavy cell wall structures made of silica or cellulose, and thus most of the entire biomass is useable. Further, cryptophyte cells can be broken and processed more easily than diatoms or dinoflagellates for commercial applications (Scholz et al., 2014). Cryptophytes from the TPG (*Teleaulax*/*Plagioselmis*/*Geminigera*) and RHO (*Rhodomonas*/*Rhinomonas*/*Storeatula*) clades have been suggested as possible species for biotechnological purposes in the areas of health improvement, solar energy exploitation, and aquaculture (Lee et al., 2019). The aim of this review is to summarize the promising microalgal compounds, with special emphasis on compounds derived from cryptophyte algae. These compounds could be useful in nutraceuticals and in medical and pharmaceutical applications for producing natural drugs and other biomedical materials.

## Methodology

Four databases, i.e. PubMed, Sciencedirect, MDPI and ResearchGate, were used in the search for relevant studies. Search words were: “cryptophytes,” “algal bioactive compounds,” “cryptophyte pigments,” “cryptophyte carbohydrates,” “cryptophyte vitamins,” “cryptophyte phytosterols,” “cryptophyte polyphenols” and “cryptophyte MAAs.” There was no time limitation because of the scarce literature about cryptophytes. Of the received hits only basic information on the bioactive compounds, and their applications in medicine and pharmacology were selected to write this review article.

## Bioactive Compounds of Cryptophytes

### Fatty Acids

Fatty acids are carboxylic acids with long aliphatic chains, which are either branched or straight, and can be saturated or unsaturated. Depending on the number of double bonds, FAs are categorized as monounsaturated FAs (MUFAs, with one double bond), or polyunsaturated FAs (PUFAs, with ≥2 double bonds). Moreover, PUFAs are classified as omega-3 (ω-3) or omega-6 (ω-6) fatty acids based on the position of the first double bond from the methyl end. In algae, the fatty acid carbon skeleton mostly varies from C12 to C24 with one or more double bonds. A wide range of FAs and their oxidized products of nutritional and chemo-taxonomic importance are found in algae, but their FA profiles are species dependent, i.e. FA production is genetically determined (Kumari et al., 2013).

Omega-3 and omega-6 fatty acids – especially eicosapentaenoic acid (EPA, 20:5 ω-3) and docosahexaenoic acid (DHA, 22:6 ω-3) are vital for normal cell activities. However, most consumers, including humans, cannot synthesize these essential long-chain PUFAs (LCPUFAs) themselves, and their capability of bioconversion is very limited. Thus, EPA and DHA need to be obtained from the diet (Burdge and Calder, 2005). Due to their biologically essential role, omega fatty acids have entered the biomedical and nutraceutical fields, where they are being used for treating various ailments such as obesity, cardiovascular diseases (CSD), arrhythmia, strokes, high blood pressure, dementia, asthma, and improving renal diseases and rheumatoid arthritis (Ryckebosch et al., 2012). For example, high consumption of EPA and DHA restricts the metabolites of arachidonic acid (AA, 20:4 ω-6) and inhibits inflammation. In addition, a balanced ω-6/ω-3 ratio is one of the most essential dietary agents to prevent obesity (Simopoulos, 2016). Omega fatty acids play an important role in normal fetal brain development and growth of infants (Jubies et al., 2012). The amount of EPA and DHA in the bloodstream of children with autistic spectrum disorders or attention deficit hyperactivity disorder (ADHD) had been lower than in control children (Calder, 2018). Deficiency of ω-3 can lead to dry skin, fatigue, heart conditions, poor memory and even schizophrenia (Pawelczyk et al., 2016; Andrade et al., 2018). According to the study of (Sanchez-Villegas et al., 2018), moderate intake of omega-3 PUFA can effectively preserve against depression irrespective of the presence of cardiometabolic disturbances, sex differences or life-style habits. Therefore, EPA supplementation is suggested as a vital anti-depressant treatment. Supplementation studies using omega-3 have indicated the decline in mortality due to fewer sudden cardiac deaths from reduction of arrhythmogenesis (Martins et al., 2013; Appleton et al., 2015; Maki et al., 2017). EPA functions as a precursor for substances like prostaglandin-3, thromboxane-3, and leukotriene-5 group. Further, EPA takes part in our defense system against inflammation by neutralizing the pro-inflammatory function of other similar molecules. Another remarkable merit of EPA is its ability to prevent clots from forming in the blood, which results in improvement of heart health, blood circulation and decreased risk of thrombosis (Gray and Bolland, 2014). DHA with antioxidant activities is the most valuable fatty acid for brain health; it helps the cognition and connection between neurons, and has beneficial aspects related to our mind including attention, imagination, memory, reasoning and judgment (Andrade et al., 2018). When alpha-linolenic acid (ALA, 18:3 ω-3) and linoleic acid (LA, 18:2 ω-6) values are less than 0.5% of energy, this can lead to impaired barrier function and wound healing as well as poor neurological and visual development in infants (Bird et al., 2018). From the ω-6 fatty acids, gamma-linolenic acid (GLA, 18:3 ω-6) is an essential fatty acid presenting anti-inflammatory properties. Arachidonic acid (AA) can be effective in controlling neurological diseases such as Alzheimer’s disease (Rapoport et al., 2007) and autism (Bell et al., 2004), and can play significant roles in muscle development - especially for individuals practicing physical exercise (Standley et al., 2013). However, the proportion of ω-6 FAs is too high in the western diet, which poses several negative health consequences. The balance of ω-6/ω-3 FA is important in reducing the risk for coronary heart disease, and is beneficial to bone health and skeletal growth (Simopoulos, 2008).

Thus far, fish have been the main source of essential LCPUFAs for humans. Alternative sustainable sources for the LCPUFAs are necessary to fulfill the need of the growing human population, since the marine fishing industry has reached its maximal production capacity. As fish do not have efficient enzymatic mechanisms for the synthesis of LCPUFAs, they accumulate these in their bodies through the consumption of microalgae, which are the principal producers of the healthy FAs (Ghosh et al., 2015). Thus, microalgae which contain approximately 30% of lipids are very attractive as natural replacements for fish and fish oil food supplements for humans (Andrade et al., 2018). Moreover, fish oil is inappropriate for some people who have fish allergies, for vegetarians, and for those who may dislike fish oil due to its possible unpleasant odor or the concerns for lipid-soluble environmental pollutants (Cuellar-Bermudez et al., 2015). Thus, supplementary products made from microalgae can be superior over the currently widely used fish oil (Ward and Singh, 2015). However, only certain microalgae can synthesize EPA and DHA and can therefore be used for commercial LCPUFA production.

One of the microalgal groups that are high in PUFA is cryptophytes. In fact, all cryptophytes regardless of the species have been shown to be rich in EPA (C_20_H_30_O_2_) or DHA (C_22_H_32_O_2_) and other ω-3 PUFAs, i.e. alpha-linolenic acid (ALA, 18:3 ω-3, C_18_H_30_O_2_) and stearidonic acid (SDA, 18:4 ω-3, C_18_H_28_O_2_) (Table 1) (Barreira et al., 2015). However, compared to marine cryptophytes, freshwater species contain less DHA (Patil et al., 2007). At the species level, for example, *Chroomonas mesostigmatica* has been introduced as promising strain for EPA extraction, whereas *Storeatula major* has shown promise for both EPA and DHA production (Peltomaa et al., 2018). In addition to ω-3 PUFAs, cryptophytes also produce ω-6 PUFAs, which are beneficial especially for dietary products (Huerlimann et al., 2010).

**TABLE 1:** Cryptophyte species with high amounts of ω-3: ALA (alpha-linolenic acid), SDA (stearidonic acid), EPA (eicosapentaenoic acid) and DHA (docosahexaenoic acid).

Species	FA (% of total)			
	ALA	SDA	EPA	DHA
*Chroomonas salina*	10.8	30.3	12.9	7.1
*Cryptomonas* sp.	25.1	30.7	12.0	6.6
*Rhodomonas* sp.	25.2	22.6	8.7	4.6
*Chroomonas mesostigmatica*	13.5	17.4	20.5	1.7
*Guillardia theta*	56.7	25.5	19.9	3.0
*Hemiselmis* sp.	53.2	20.5	21.2	5.1
*Proteomonas sulcata*	58.5	16.2	12.7	12.6
*Storeatula major*	41.9	32.1	16.0	10.0
*Teleaulax acuta*	46.2	13.4	26.0	14.3
*Teleaulax amphioxeia*	43.3	20.5	23.6	12.7

(adapted from Barreira et al., 2015; Patil et al., 2007).

### Sterols

Sterols are an important family of lipids that are biosynthesized by all eukaryotic organisms (Desmond and Gribaldo, 2009). Cholesterol, the prominent sterol in animals, is scarcely found in plants. Alternatively, plants are composed of certain types of phytosterols, which are functionally and structurally similar to cholesterol (Hernandez-Ledesma and Herrero, 2014). Unlike cholesterol, humans have to obtain phytosterols from their diet since they cannot produce them endogenously (Tasan et al., 2006). Up to now, higher plants have been the major industrial source of phytosterols (Fernandes and Cabral, 2007), but phytosterols are also found in algae (Hernandez-Ledesma and Herrero, 2014). Sterol distribution in microalgae presents a large number of structures that reflect distinct differences in sterol biosynthetic pathways (Nes, 2011). Sterol compound differs according to the algal strain, and can be modified by temperature, light intensity and growth phase. Together these features make microalgae a potential and promising source of phytosterols for health benefits (Galasso et al., 2019). Since phytosterols can act as secondary messengers, similar to hormones, they affect cellular processes including neurotransmission and development (Francavilla et al., 2010). Phytosterols derived from microalgae have been shown to have anti-cancer, anti-inflammatory, antioxidant or anti-cholesteroligenic (Hwang et al., 2014; Cabral and Klein, 2017), immunomodulatory (Caroprese et al., 2012), anti-diabetic (Lee et al., 2004) and antibacterial properties (Luo et al., 2015). Additionally, evidence suggests that phytosterols offer protection against nervous system disorders like Alzheimer’s disease and autoimmune encephalomyelitis (Ahmed et al., 2015). The phytosterols derived from microalgae can decrease the dietary cholesterol absorption and thus prevent hypercholesterolemia (Chen et al., 2014; Luo et al., 2015). By becoming incorporated into the cell membrane, phytosterols can alter the activity of some membrane-bound enzymes and the signal transduction in pathways that cause tumor growth (Lopes et al., 2013). Further, algae-derived phytosterols have been shown to have anti-diabetic activity in diabetic rats, suggesting that they could have potential in the prevention of type 2 diabetes in humans (Lee et al., 2004).

Five different phytosterols including crinosterol (C_28_H_46_O), brassicasterol (C_28_H_46_O) (the major sterol in cryptophytes), β-sitosterol (C_29_H_50_O; BS) campesterol (C_28_H_48_O) and stigmasterol (C_29_H_48_O) have been found in cryptophytes (Table 2) (Taipale et al., 2016; Peltomaa et al., 2017). BS possesses a skin conditioning influence used in anti-aging cosmetic products, moisturizer, sunscreen and body wash (Han et al., 2014). BS also plays a crucial role in modulating antioxidant enzymes and human estrogen receptor (Song et al., 2000), as well as in blood vessel formation, thus having wound healing potential (Moon et al., 1999). Moreover, BS has been used in the treatment of hyperlipidemia, and has antipyretic effects and immune-modulating activities in HIV-infected patients (Sayeed et al., 2016). While crinosterol and brassicasterol are used as anti-aging factors (Sun et al., 2014), stigmasterol is often regarded as the most valuable phytosterol due to its anti-inflammatory effects and health-promoting benefits (Gabay et al., 2010; Tang et al., 2011). Benefits of stigmasterol have been shown in the therapy of rheumatic diseases as an anti-stiffness factor; it also has noticeable anti-osteoarthritic and anti-catabolic features (Gabay et al., 2010).

**TABLE 2:** Bioactivities of phytosterols derived from cryptophytes.

Identified phytosrerols	Cryptophytes species	Phytosterol content (µg/mg dry weight)	Biological activity
Crinosterol	*Chroomonas mesostigmatica*	0.93	Anti-aging
	*Hemiselmis* sp.	0.43	
	*Rhodomonas salina*	0.14	
	*Storeatula major*	0.24	
	*Teleaulax amphioxeia*	0.45	
Brassicasterol	*Chroomonas mesostigmatica*	0.02	Cholesterol
	*Cryptomonas ovata*	—	Lowering
	*Rhodomonas minuta*	—	Anti-aging
	*Guillardia theta*	0.31	
	*Hemiselmis* sp.	1.11	
	*Proteomonas sulcata*	0.71	
	*Rhodomonas salina*	0.84	
	*Storeatula major*	0.72	
	*Teleaulax acuta*	0.35	
Stigmasterol	*Storeatula major*	—	Thyroid-inhibitory
	*Guillardia theta*	0.36	Antioxidant
	*Cryptomonas ovata Rhodomonas minuta*	—	Hypoglycaemic
		—	Cholesterol-lowering
			Anti-cancer
			Anti-inflammatory
			Anti-osteoarthritic
Campesterol	*Cryptomonas marssonii*	—	Cholesterol-lowering
			Anti-cancer
			Anti-angiogenic
			Antioxidant
β-Sitosterol	*Cryptomonas marssonii*	—	Anti-cancer
			Anti-inflammatory
			Analegesic activity
			Antihelminthic
			Antimutagenic

(adapted from Luo et al., 2015; Peltomaa et al., 2018).

### Carotenoids

Carotenoids are considered as the most varied and extensive pigments which are found in nature. They are lipid soluble carbon compounds with a common C40 backbone structure of isoprene units (terpenoid). They are classified into two groups: carotenes (hydrocarbon carotenoids, like β-caroten and lycopene) and xanthophylls (oxygenated carotenoids, such as lutein, zeaxanthin and astaxanthin) (Gong and Bassi, 2016). Thus far, 600 different carotenoids have been identified that have various biological activities in algae, bacteria, plants and animals (Polivka and Sundstrom, 2004).

Many of the effective medical and nutritional studies show that the antioxidant properties of carotenoids can play a remarkable role in decreasing the prevalence of many diseases; specifically those affected by light (Cardozo et al., 2017), as carotenoids directly create photoprotection against UV light in the skin (Aust et al., 2005). Since carotenoids show antioxidant benefits and nutritional value for hair and skin, they are applied as effective ingredients with biological functions in cosmetics such as creams and lotions (Stahl and Sies, 2012). The benefits that carotenoids offer to human health are lower risk of inflammation, heart disease and type 2 diabetes, cancer prevention, improved eye health and protection of neurons (Novoveska et al., 2019). According to some reports, a diet rich in carotenoids is connected to a reduced risk of various kinds of cancers such as lung and stomach, ocular diseases (eye diseases) like cataract and age-related macular degeneration (AMD) and cardiovascular diseases (Krinsky and Johnson, 2005; Moeller et al., 2006). For example, astaxanthin shows anti-hypertensive properties and can influence the reduction of blood pressure and heart strokes in rats, whereas β-carotene could prevent the activation and nuclear translocation of transcription factors (Sathasivam and Ki, 2018). The use of synthetic antioxidants in the European Union countries is under strict regulation, because of their possible potential health risks. Thus, natural antioxidants can be used as safe alternatives in the industry (Gouveia et al., 2010). The increasing interest for natural and organic beauty products boosts the commercial potential for carotenoids extracted from microalgae.

Cryptophytes have carotenoids that are useful in different industries, specifically in medicine and pharmacy. The major carotenoid in cryptophytes is called alloxanthin (C_5_H_4_N_4_O_2_) (Ansotegui et al., 2003; Cunningham et al., 2018) (Table 3), and is present e.g. in the marine cryptophytes *Teleaulax acuta* and *Hemiselmis* sp. (Seoane et al., 2005). Cryptophytes also contain other kinds of carotenoids, including α-carotene (C_40_H_56_) (Table 3), crocoxanthin (C_40_H_54_O), monadoxanthin (C_40_H_54_O_2_), cryptoxanthin (C_40_H_56_O) (Margulis and Chapman, 2009), lutein (C_40_H_56_O_2_) and lycopene (C_40_H_56_). The amount of α-carotene in *Cryptomonas* sp. and *Hemiselmis virescens* has been 0.28 and 0.1 mg/g dry weight respectively (Allen et al., 1964). Cryptoxanthin, which is another interesting carotenoid of cryptophytes, is intimately connected to β-carotene in terms of structure, with only an addition of a hydroxyl group. Cryptoxanthin is a member of carotenoids class, which are known as xanthophylls. In the human body, cryptoxanthin is converted to vitamin A (retinol) and is hence called provitamin A. Like other carotenoids, it is an antioxidant and can help to block free radical damage to cells and DNA, as well as stimulate the repair of oxidative damage to DNA (Lorenzo et al., 2009). Recent studies suggest that β-cryptoxanthin (3-hydroxy-β-carotene) could conceivably play as a chemopreventive factor against lung cancer (Lian et al., 2006). Similarly, α-carotene exhibits anti-carcinogenic and anti-diabetic activities (Sathasivam and Ki, 2018). Lutein and its derivatives are found only in red algae (mainly macroalgae), cryptophytes, euglenophytes, chlorarachniophytes and green algae (Takaichi, 2011). It accumulates preferentially in the macula lutea (area of the retina near the optic disk that provides central vision), protecting the retina from oxidative damage from UVR. Lutein can also improve skin elasticity, and has antioxidant, anti-inflammatory, photoprotection and anti-carcinogenic activities (Woodside et al., 2015). Lycopene is a rare algal carotene, identified by visible and mass spectrometry and cochromatography as a trace constituent in *Cryptomonas ovata* (Pennington et al., 1985). The biological functions of this composition include photoprotection and radioprotection against gamma-radiation-induced cellular damages. It is also a strong antioxidant with antiradical activity. According to (Kong et al., 2010), lycopene performs a principal role in chronic diseases including cardiovascular disease, neurodegenerative disorders, cancer and atherosclerosis.

**TABLE 3:** Cryptophyte species with the carotenoids α-carotene and alloxanthin

Strains	α-carotene (pg cell^−1^)	Alloxanthin (pg cell^−1^)
*Chroomonas* sp.	6.2	9.8
*Cryptomonas acuta*	6.9	7
*Cryptomonas irregularis*	5.9	6.4
*Cryptomonas ovata*	2.7	2.8
*Cryptomonas curvata*	5.6	7.6
*Rhodomonas falcata*	3.5	3.6
*Rhodomonas salina*	0.5	0.5
*Storeatula* sp.	4.1	4

(adapted from Cunningham et al., 2018).

### Phycobiliproteins

**Figure 3:**
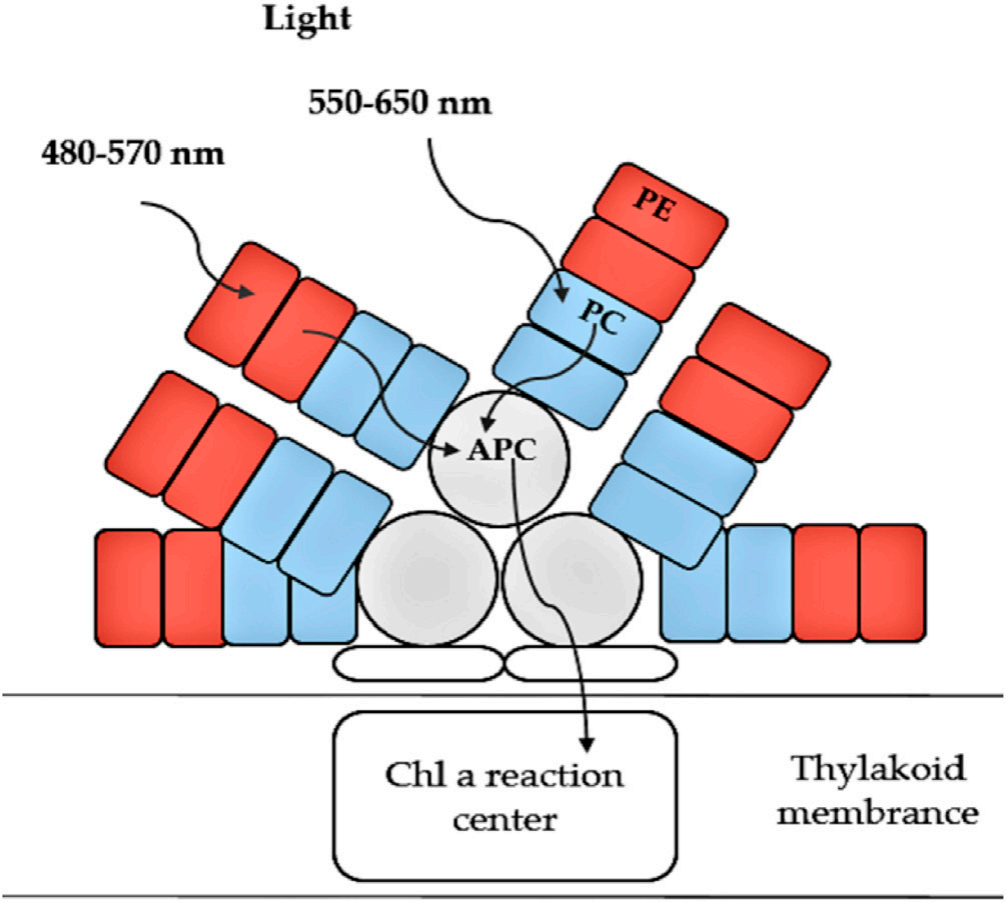
Schematic structure and function of phycobiliproteins in light-harvesting. PE, Phycoerythrin; PC, Phycocyanin and APC, Allophycocyanin (adapted from Heldt et al., 2011).

Phycobiliproteins (PBPs) are a group of colored proteins that are located in phycobilisomes (PBS), and act as photosynthetically active pigments. They can be easily extracted as pigment-protein complexes (Figure 3). PBPs are classified into three groups in accordance with the existence of diverse chromophores (Ducret et al., 1998): 1) phycoerythrin (PE: λmax 480–570 nm); 2) phycocyanin (PC: λmax 590–630 nm) and 3) allophycocyanin (APC: λmax 620–665 nm). Recently, numerous studies have discovered bioactivities of different phycobiliproteins showing several antioxidant and radical scavenging activities, as well as anti-inflammatory and anti-cancer activities (Stengel et al., 2011; Ravi et al., 2015; Jiang et al., 2017). PBPs include aspects relevant in human medicine including antimicrobial, neuroprotective and hepatoprotective properties (Richa et al., 2011). PBPs can provide great protection to kidney cells against oxidative stress and cellular damage created by mercuric chloride HgCl_2_ (Ughy et al., 2015). They play a substantial role in the commercial sector, as they have several applications. PBPs are widely used as natural pigments in numerous food and cosmetic industry products such as jellies, dairy products, chewing gum (Santiago-Santos et al., 2004), lipstick, sun-protecting cream and eye shadow pallets (Sonani et al., 2016). A number of studies suggest that PBPs also have health promoting abilities, and may therefore provide a range of pharmaceutical applications related to e.g. their anti-aging, anti-Alzheimer and anti-cancer activities (Batista et al., 2006; Sonani et al., 2016). Phycoerythrin has been reported to have antifungal, antibacterial, antioxidant and dermatoprotective activities (Verma et al., 2018), whereas antibacterial, immune system modulating, anti-cancer (prostate, breast and cervix), melanogenesis inhibiting and hematological roles have been reported for PC (Soni et al., 2015). Additionally, the consumption of edible algae containing PC has health promoting activities including prevention of inflammation, degradation of plasma lipid concentration through reduction in cholesterol absorption and inhibition of oxidative stress via blocking lipid peroxidation (Ku et al., 2013). In hamsters that were fed a diet supplemented with PC, fatty lesion development and cardiac production of superoxide anion were considerably reduced (Riss et al., 2007).

**Figure 4:**
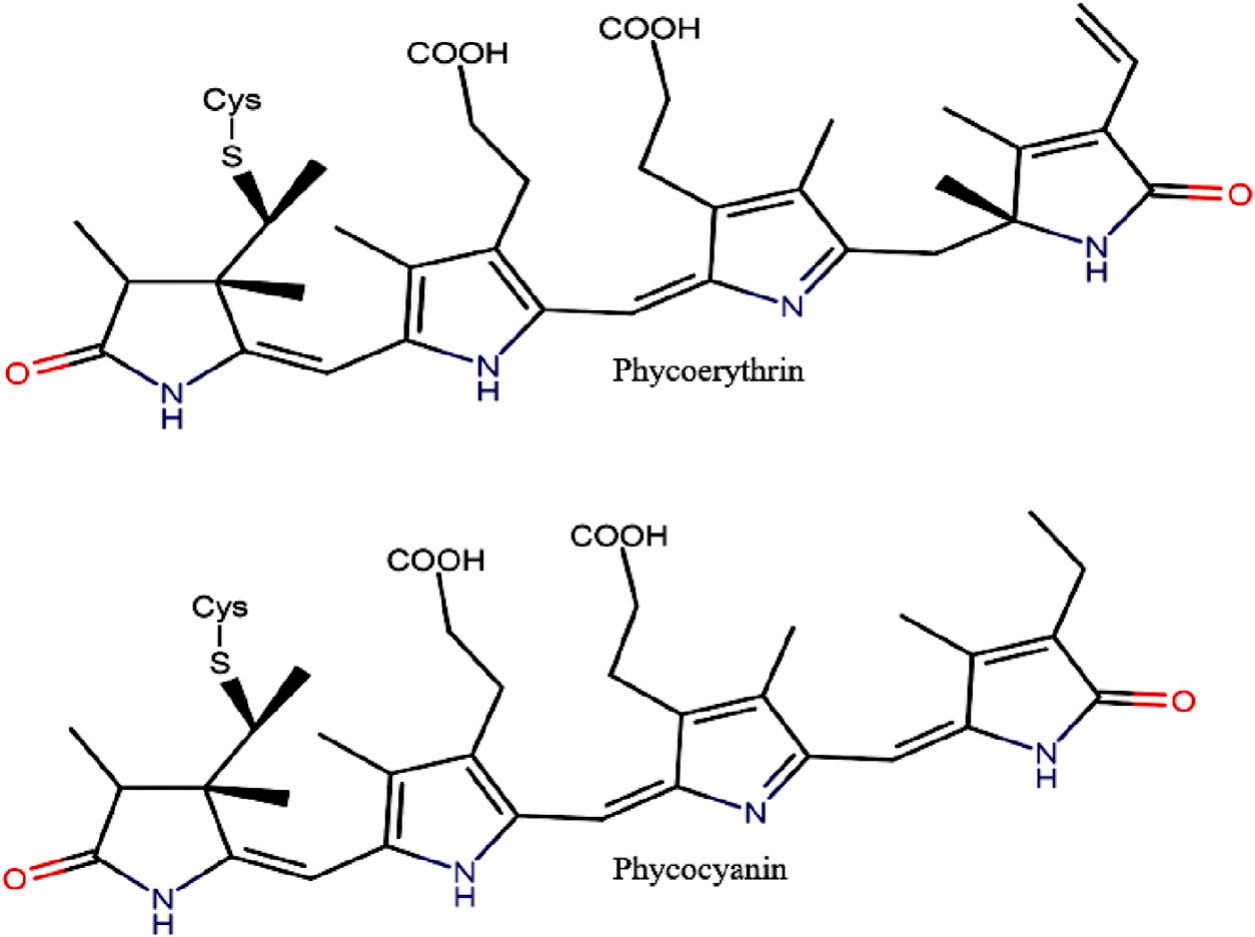
Chemical structure of PE and PC (adapted from Wilk et al., 1999; Hoseini et al., 2013).

PBPs are the major light-harvesting pigments of cryptophytes (Sidler, 1994). As cryptophytes contain one biliprotein, either PC or PE (Figure 4) and no APC, the path of energy transfer is different from red algal and cyanobacterial phycobiliproteins; in the absence of allophycocyanin in cryptophytes, chlorophyll C_2_ acts as an intermediate between the biliprotein and chlorophyll a (Hill and Rowan, 1989). The cryptophyte biliproteins are named based on different wavelengths and their respective absorption maxima (e.g. phycoerythrin 545−PE545 and phycocyanin 630−PC630) (Table 4).

**TABLE 4:** Classification of cryptophytes based on biliprotein type and PBP concentration.

Genus	Biliprotein Type	PBP (pg cell^−1^)
*Cryptopmonas*	PE565 or none	2.3–40.4
*Rhodomonas*	PE545	2.6–13.9
*Rhinomonas*		3.3
*Storeatula*		14.6
*Guillardia*		0.9
*Hanusia*		1.9
*Plagioselmis*		—
*Teleaulax*		—
*Geminigera*		6.6
*Proteomonas*		1.2–10.3
*Hemiselmis*	PC615, PC630	0.2–1.6
	PC577 or PE555	0.5
*Chroomonas*	PC630 or PC645	6–12.2
*Komma*	PC645	—
*Flacomonas*	PC569	—

(adapted from Tanifuji & Onodera, 2017; Cunningham et al., 2018).

The marine cryptophyte *Rhodomonas* and the fresh water cryptophyte *Cryptomonas* are promising candidates for the production of PE, a red-colored PBP used as a fluorescent probe and analytical reagent, as well as a natural dye in food, beauty products and cosmetics (Chaloub et al., 2015; Cunningham et al., 2018). Moreover, the genus *Chroomonas* has been reported as a great source of PC (Cunningham et al., 2018), a blue light-harvesting phycobiliprotein applied as colorant in cosmetic and with antitumor, antioxidant and anti-inflammatory activities in medicine (Liu et al., 2016). Compared with other phycobilisome containing algae, such as red algae and cyanobacteria, a significant advantage of cryptophytes is the presence of only one type of biliprotein in one species. This, together with the lack of a cell wall, makes the unit functions associated with cell disruption and downstream processing of PE easy and economically feasible (Chaloub et al., 2015).

### Mycosporine-like Amino Acids

**Figure 5:**
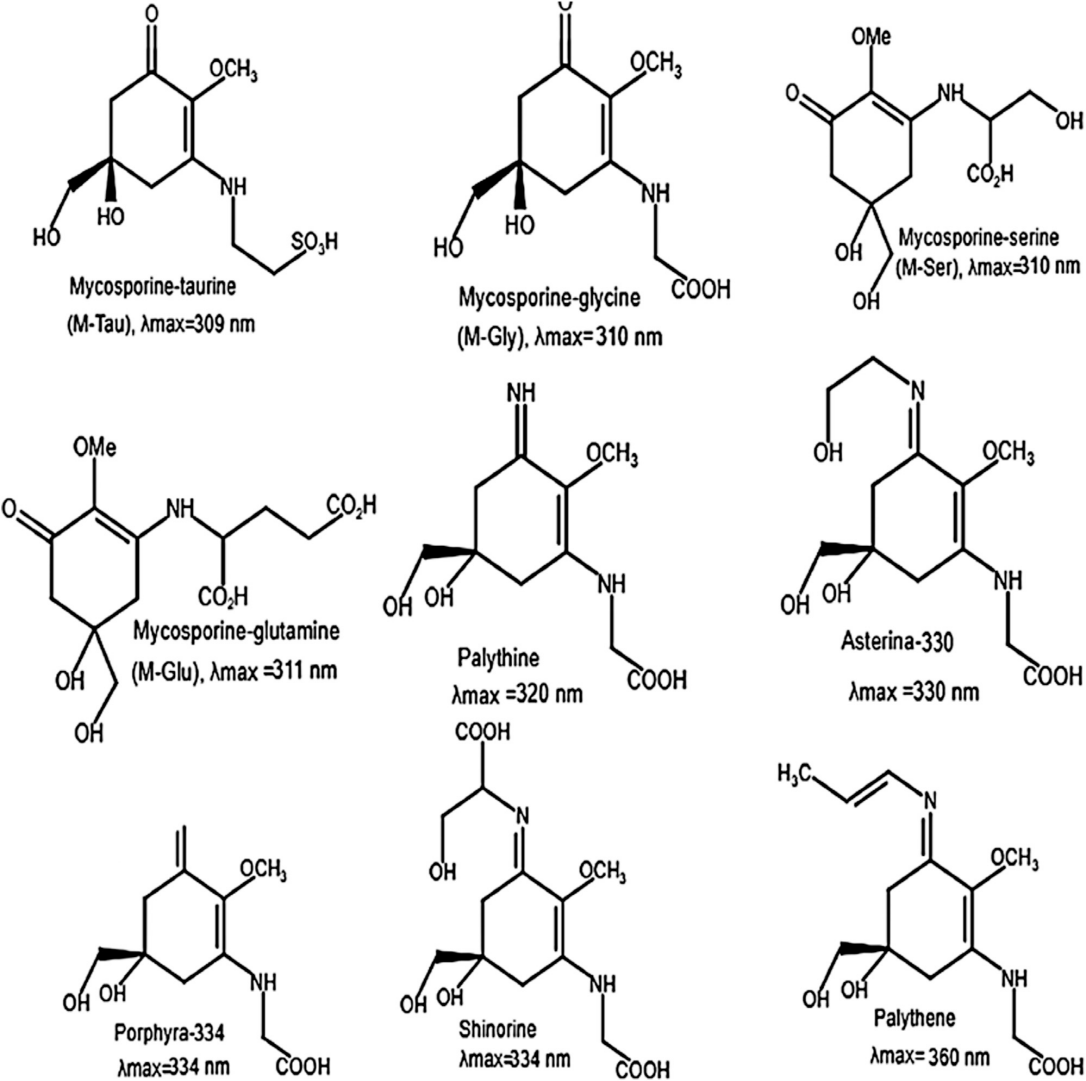
Chemical structure of some MAAs and their maximum absorption (λ_max_) (adapted from Chrapusta et al., 2017).

MAAs are a family of intracellular compounds protecting aquatic organisms against solar radiation. These UV-absorbing compounds are water soluble and low molecular weight components (<400 Da). Their chemical structure is based on either a cyclohexenone (wavelength maxima (λ_max_) 310 nm in ultraviolet-B) or cyclohexenimine (λ_max_: 360 nm in ultraviolet-A) ring structure with amino acid substituents (Karentz et al., 1991). Biosynthesis of MAAs occurs via a branch of the shikimic acid pathway. Thus far, 20 MAAs have been identified from different organisms (Carreto and Carignan, 2011), of which some examples are presented in Figure 5. They are present intracellularly in many marine and freshwater organisms (Rezanka and Temina, 2004). Although other marine organisms obtain MAAs by diet and bacterial association, algae biosynthesize MAAs themselves (Carroll and Shick, 1996).

In addition to their role as a sunscreen, MAAs act as antioxidants (Dunlap and Yamamoto, 1995). Skin pigmentation is an endogenous and protective structure against the damages resulted from high exposure to sunlight, since melanin absorbs a broadband of UV-radiation and removes one of the main UV-induced cellular subsequences, reactive oxygen species (ROS) (Brenner and Hearing, 2008). A large number of UV filters are produced around the world yearly due to the consumer demand for sunscreen in lotions, lipsticks, moisturizers and facial makeup. Considering the possible impact of MAAs on the skin, a study including 20 middle-age women reported that a cream containing 0.005% MAAs obtained from red algae can counteract UV-A effects and develop skin smoothness (Morone et al., 2019). Mycosporine-glycine has been reported to have proper antioxidant activity, providing some preservation against photooxidative stress derived by ROS (Cardozo et al., 2017). Additionally, MAAs are regarded as anti-cancer factors because of their anti-proliferative activities on neoplastic cells, and their antioxidant activities involved in the suppression of tumor proliferation (Chrapusta et al., 2017). The anti-photoaging role of MAAs has been examined; based on *in vitro* analysis; asterina-330 can significantly decrease the lipid peroxidation, which affects initiating and mediating of the aging process (Coba et al., 2009). Moreover, porphyra-334 shows inhibitory potential on the UV-increased activity of elastase leading to elastin decomposition and wrinkle formation (Ryu et al., 2014). The microalgal-derived shinorine, mycosporine-glycine and porphyra-334 exhibit inhibitory effects on the expression of inflammation-related genes, hence showing anti-inflammatory potential (Rosic, 2019).

The photoprotective UV filtering and antioxidant role of MAAs have also been supported by affirming the high photostability and the release of heat to the medium as the leading pathway of the photoexcited molecules (Conde et al., 2007). The most comprehensive study (152 algal species) on MAAs in microalgae reported that high amounts of these compounds are found in dinoflagellates, cryptophytes, prymnesiophytes and raphidophytes (Jeffrey et al., 1999; Rezanka and Temina, 2004). In the study of (Llewellyn and Airs, 2010), *Rhodomonas baltica* possess MAAs compounds with high levels at 310 nm; the λ_max_ at 310 nm is consistent with structures of mycosporine-glutamine (M-Glu, C_13_H_19_NO_8_), mycosporine-taurine (M-Tau, C_13_H_19_NO_8_), mycosporine-serine (M-Ser, C_10_H_17_NO_7_S) and mycosporine-glycine (M-Gly, C_10_H_15_NO_6_). M-Gly has been reported to have antioxidative, anti-inflammatory and antiaging activities (Suh et al., 2014; Ngoennet et al., 2018). M-Gly purified from macroalgae *Porphyra yezoensis* has considerable effect on the wound healing process in humans (Choi et al., 2015). Additionally, M-Tau with antioxidant activity exhibits efficient protective ability toward cell damaged by ROS (Zhang et al., 2007). These provide new insights into the application of mycosporine-like amino acids in the cosmetic sectors.

### Polysaccharides

Polysaccharides, especially sulfated exopolysaccharides (EPS), form a group of important high molecular weight biopolymers released from microorganisms like microalgae into the environment during their growth (Liu et al., 2016). Evaluation of structures, compositions, functions and characteristics of EPS are necessary for understanding their production mechanism and attributes for promising applications. The primary compositions of EPS contain lipids, polysaccharides, nucleic acids (DNA) and proteins. Various factors such as nutrient availability, strain, species and physiology affect diversity of polymers in EPS and the numbers of particular compounds (Xiao and Zheng, 2016). They act as antiviral factors, health foods and antioxidants. They present anti-inflammatory properties, drag-reducing substances and play a considerable role in the immunomodulatory system (Raposo et al., 2013).

Most algal polysaccharides (agars, carrageenans, alginates) used in different industries are gained from macroalgae. However, it has been shown that the polysaccharides from some marine microalgae show antiviral bioactivity against various kinds of viruses, including mammalian viruses (Radonic et al., 2010). Investigations of sulfated polysaccharides (sPS) from marine microalgae, especially ones produced by the red microalga *Porphyridium*, report the antiviral activity of sPS. The mechanisms of activity are not yet entirely understood, but can relate to the anionic nature of the sPS. Sulfate polysaccharides are able to prohibit infection by different viruses via preventing infiltration of viral particles into host cells. However, there are also other mechanisms, such as the restriction of binding/adsorption, or even duplication throughout the early phases of the virus cycle, which may be involved in the antiviral activities of sPS (Raposo et al., 2014).

In addition to their function as dietary fiber, sulfated polysaccharides secreted from microalgae have the ability to protect systems against oxidative and radical stress factors by prohibiting the activity and accumulation of reactive chemical species and free radicals (Sun et al., 2009). Polysaccharides from marine microalgae, including *Porphyridium, Phaeodactylum* and *Chlorella stigmatophora*, have shown pharmacological attributes, like anti-inflammatory effects, and function as immunomodulatory factors. Studies have proven the direct stimulating significance of *Phaeodactylum tricornutum* on the immune cells by the positive phagocytic activity (Guzman et al., 2003). One notable feature of polysaccharides is the potentiality to suppress tumor cell growth. The homopolysaccharide of *Gymnodinium impudicum* with its immunomodulatory properties prevented the growth of tumor cells, both *in vitro* and *in vivo* (Yim et al., 2004). In a recent study (Gardeva et al., 2014), intense anti-tumour activity has been reported by the polysaccharide of *Porphyridium cruentum*. This sulfated polymer effectively controlled Graffi myeloid tumor division *in vitro* and *in vivo*. Polysaccharides have immunostimulating effects that cause inhabitation of tumor cell activity. For example, EPS from *Porphyridium* has potential as an anticancer agent that inhibits the growth of different cancer cell lines (Gardeva et al., 2014). EPS from unicellular algae are also considered as possible candidates in reducing coronary heart disease because of their hypocholesterolaemic effects (Dvir et al., 2009), anti-adhesive and anti-inflammatory activities, prevention of tumor cell growth and immunomodulatory effects (Raposo et al., 2015). The production of sulfated exopolysaccharides from the red microalga *P. cruentum*, the cyanobacterium *Spirulina*, and the cryptophyte *Chroomonas* have already shown potential for commercial exploitation (Nie et al., 2002; Bermudez et al., 2004; Keidan et al., 2009).

Reports of EPS production and characterization of cryptophytes is rare. There is a handful of articles on this topic, and only few of them show profiling results. However, the profiling of the EPS secreted by a tropical cryptophyte, *Cryptomonas tetrapyrenoidosa*, has been made for two different fractions isolated by anion exchange chromatography (Table 5) (Giroldo et al., 2005). In that study, Fraction 1 eluted with 0.5 M NaCl while Fraction 2 eluted with 1.0 M NaCl. Fraction 1 had fucose, galactose, N-acetyl glucosamine and mannose as the main components, whereas galactose and glucuronic acid were the main EPSs in Fraction 2. Additionally, the EPS of *Cryptomonas obovata* (also a tropical strain) has been described (Table 5) (Giroldo et al., 2005). The EPS profiles were generally similar between the two strains, but the proportions were quite distinct. The main EPS of *C. obovata* was the sulfated fucose-rich polysaccharide; this strain was also rich in N-acetyl galactosamine (GlcNAc; Table 5). Studies indicate that fucose-containing sulfated polysaccharides from algae potentially act as skin-cancer preventive factors (Ale et al., 2011) and a strong anticoagulant (Raposo et al., 2015). N-acetyl galactosamine can have cytoprotective activities to restore the integrity and normal operation of the mucous membrane in humans, and act as an inexpensive and non-toxic treatment for inflammatory bowel disease (Chen et al., 2010). Moreover, GlcNAc can improve skin health by increasing the proliferation and collagen expression of skin fibroblasts (Chen et al., 2008), in addition to its moisturizing properties (Bissett et al., 2007). Finally, polysaccharides such as rhmanose, xylose, glucose and glucuronic acid derived from these cryptophytes have had antioxidant, antibacterial, antiviral, antilipidemic, antiglycemic and infection prevention potential (Raposo et al., 2015).

**TABLE 5:** Carbohydrate composition and total polysaccharide of *C. tetrapyrenoidosa* and *C. obovata*.

	*C. tetrapyrenoidosa*		*C. obovata*
	Carbohydrate composition (%)		% Total polysaccharide
	Fraction 1	Fraction 2	
Rhamnose	9.0	0.8	15.3
Fucose	24.3	8.6	41.6
Xylose	4.7	0.4	2.7
Mannose	15.4	0.8	3.6
Galactose	13.7	36.0	4.4
Glucose	3.5	0.5	2.1
Glucuronic acid	4.1	47.0	4.3
N-acetyl galactosamine	8.6	0.27	26.9

(adapted from Giroldo et al., 2005; Giroldo & Vieira, 2002).

### Vitamins

Vitamins - vital organic micronutrients - cannot be directly synthesized by animals in sufficient quantities. Therefore, animals must gain them from external sources. These compounds are necessary for urgent metabolic functions, and act as precursors for essential enzyme cofactors (Weels et al., 2017). Microalgae are an unexplored source of almost all kinds of vitamins including pro-vitamin A (α- and β-carotene, apocarotenoids), vitamin C (ascorbic acid), vitamin E (tocopherols and tocotrienols), vitamin D, and some vitamins of the B group (particularly B_1_ and B_12_) (Uribe et al., 2017; Galasso et al., 2019). Natural and synthetic retinoids (a class of compounds including vitamin A and the main apocarotenoid produced in algae) have been mainly represented in preventing the growth and development of various sorts of tumors, including skin, breast, oral, lung, prostatic and bladder cancers (Altucci and Gronemeyer, 2001; Jonas et al., 2015). Vitamin C shows valuable health effects, such as cancer and atherosclerosis prevention, and serves as an immunomodulatory agent, for instance for the prohibition of tuberculosis (Nunes-Alves et al., 2014). According to studies, vitamin C has a significant effect on the prevention of gastric cancer (Granger and Eck, 2018). Vitamin D plays an important role in a vital process of calcium absorption and metabolism for bone health and homeostasis, and it is beneficial in cancer prevention and anti-neurodegenerative effects. This vitamin also regulates calcium and phosphate metabolism and is essential for maintaining bone health, i.e. for preventing osteomalacia and osteoporosis (Feldman et al., 2014). Vitamin E inhibits lipoprotein oxidation processes that have a role in the growth of cancer in mice. Furthermore, it improves endothelial function and reduces vascular damage (Corina et al., 2019). High levels of Vitamin B12 are attributed to reduced risk of breast cancer, and can act on DNA repair and histone methylation (Gruber, 2016).

The green microalga *Dunaliella tertiolecta* has vitamin B_12_, B_2_, E and provitamin A. Moreover, the green microalga *Tetraselmis suecica* is a potential source of vitamin B_1_, B_3_, B_5_, B_6_, and C (Fabregas and Herrero, 1990). *Chlorella* species have generally been detected to contain vitamin B_7_ in high concentrations, and around 9–18% of *Chlorella* strains have been reported to contain vitamin B_12_ (Koyande et al., 2019). *Chlorella* and *Spirulina* contain high concentrations of B_9_ (folic acid), a principal vitamin to cell formation and bone and teeth development. Further, B_9_ maintains normal metabolism and preservation of skin membranes (Becker, 2007). The amount of vitamin C varies in algae, and a study on algal species reported a significant amount of vitamin C (C_6_H_8_O_6_) in the cryptophyte *Cryptomonas maculata* (6.45 pg cell^−1^) (Brown and Miller, 1992). Thiamine (B_1_, C_12_H_17_N_4_OS+) concentration in the cryptophyte *Rhodomonas salina* has been shown to be about 358.8 nmol g cell^−1^ (Sylvander et al., 2013). However, cryptophytes are not reported to be rich in other vitamins.

### Phenolic Compounds

Phenolic compounds are secondary metabolites and, due to their high production under stress in organisms, are frequently identified as stress compounds. Phenolics have chemical protecting mechanisms against UV radiation (Coba et al., 2009) and metal contamination (Connan and Stengel, 2011). Chemically, polyphenols are classified into several classes, such as phenolic acids, flavonoids, isoflavonoids, stilbenes, lignans, and phenolic polymers (Ozcan et al., 2014).

Due to their therapeutic functions, phenolic compounds have recently gained the interest of consumers and functional food manufactures. Extracted phenolic compounds show a vast array of activities, such as anti-radical, UV-protection and anti-HIV, and they act as inhibitors of melanin formation. They also have been reported to have anti-adipogenic activities, and neuroprotective effects, and a potential treatment of Alzheimer’s disease (Stengel et al., 2011). An extensive review (Cornish and Garbary, 2010) shows the promising applications of polyphenols, including algae as antioxidants, in human health and nutrition. Food that is rich in antioxidants has been supported to prevent cardiovascular disease (CVD) that represent a multiprocess disorder including oxidative stress, inflammatory dysfunction, and vascular remodeling. A clear association between the consumption of seaweed by Japanese people and reducing risk of mortality by CVDs has also been detected (Shimazu et al., 2007). Further, polyphenols extracted from the brown macroalga *Ecklonia* sp. reduced UVB-induced skin tumor improvement in mice notwithstanding whether the polyphenols were used topically or as a dietary component, suggesting that the activity of these algae-based antioxidants is uninfluenced by digestive processes (Hwang et al., 2006). Phlorotannins, a type of tannins that are a class of astringent, polyphenolic biomolecules, have been detected to have repressive effects on HIV-1 reverse transcriptase activity, which means that they can fight against human immunodeficiency viruses (Ahn et al., 2004). They also involve in the development of anti-allergic compounds similar to phlorofucofuroeckol-B, which show an impact on histamine release (Sugiura et al., 2007), and has a protective effect against diabetes (Lee and Jeon, 2013). Additionally, they protect cells from radiation-induced injury (Shin et al., 2014), which is another indication of their efficacy in anti-oxidative protection.

Several classes of flavonoids, such as isoflavones, flavanones, flavonols, and dihydrochalcones are found in microalgae (Manach et al., 2004). Flavonoids contain a broad spectrum of health-promoting effects and are fundamental components in a diversity of nutraceutical, pharmaceutical, medicinal and cosmetic applications (Andrade et al., 2018). Flavonoid-rich foods have been shown to have about 50% reduction in the risk of dementia, a delay in the Alzheimer’s disease and decrease the risk of developing Parkinson’s disease (Vauzour et al., 2010). Accordingly, the potential pharmaceutical applications of algal polyphenols have been widely investigated because of their anti-cancer, photo-aging preventing and anti-inflammatory effects (Thomas and Kim, 2011; Li et al., 2014; Machu et al., 2015).

A specific class of flavonoids, 2-styrylchromones (2-SC, C_17_ H_12_ O_2_), was extracted from the marine cryptophyte *Chrysophaeum taylori* in 1986 by W. H. Gerwick (Gomes et al., 2010). Certain analogues of these components have been synthesized and assessed in biological systems, showing that both the natural and synthetic compounds possess a myriad of biological activities (Table 6) including anti-allergic, anti-tumor (Li et al., 2007), antioxidant, antimicrobial, antiviral, anti-inflammatory (Madhava Rao et al., 2016), antifungal, hepatoprotective (Pinto et al., 2015), anti-infective, immune system promoting and skin protective from UV radiation (Gomes et al., 2010; Tungmunnithum et al., 2018). This clearly demonstrates the ability of cryptophytes to produce complex phenolic compounds. Characterization, recognition and exploration of phenolic compounds in microalgae is indispensable, specifically since they may possess unique phenolic compounds (Safafar et al., 2015).

**TABLE 6:** Some biological activities of 2-styrylchromones.

Biological activity	Specific effect (s)	Chemical structure
Antiallergenic	Inhibition of histamine release from passively sensitized rat peritoneal cells	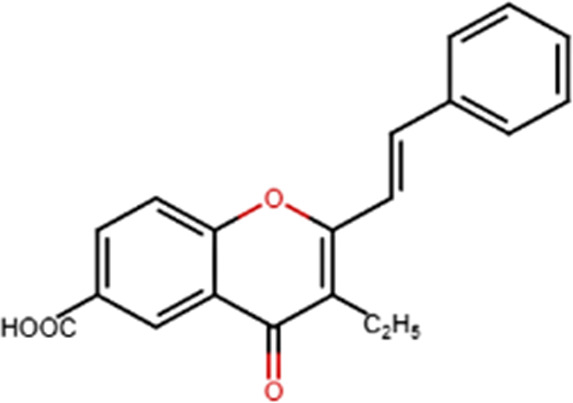
Antitumor	Cytotoxicity to tumor cell lines	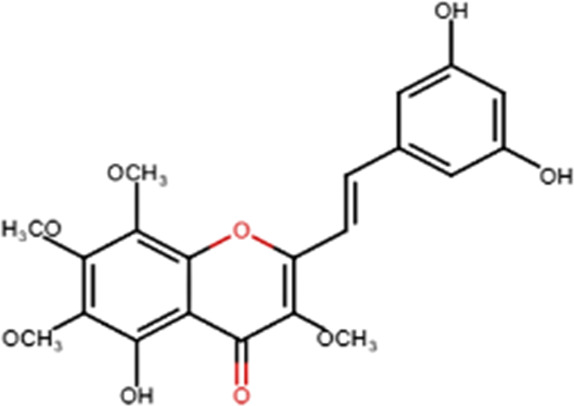
	Tumor-specific cytotoxic effect and tumor-specific antiproliferative effect	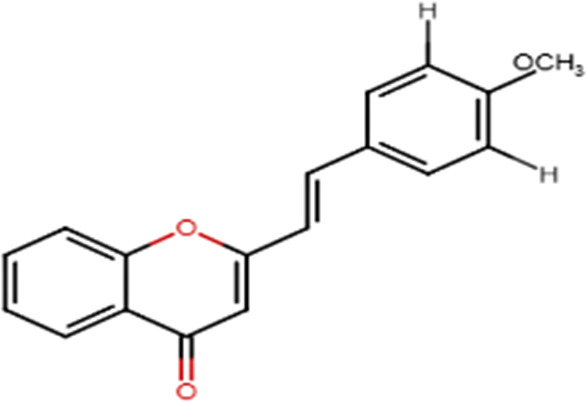
	Antiproliferative effect against human carcinoma cell lines	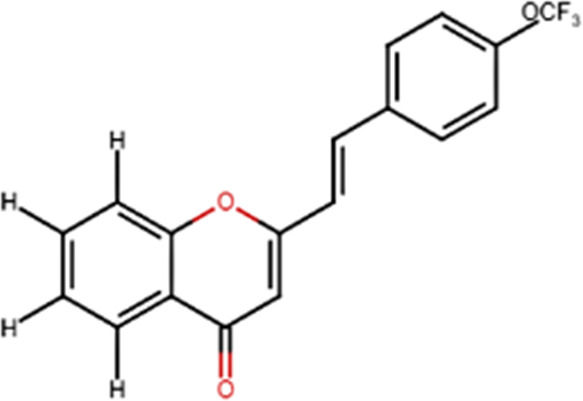
Antiviral	Activity against human rhinoviruses (HRV)	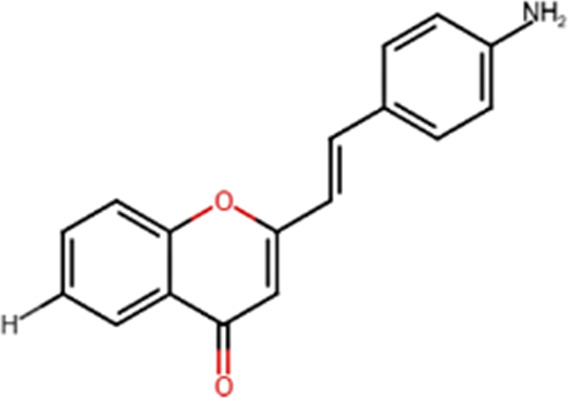
Antioxidant	Protective activity against the tert-butylhydroperoxide from proxidant hepatotoxicity in rat hepatocytes and scavenging impact of ROS and reactive nitrogen species	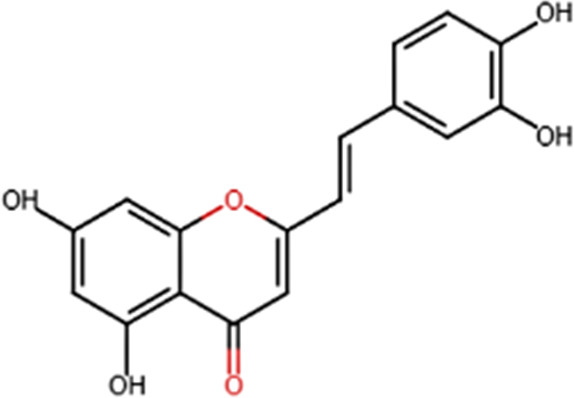
Anti-inflammatory	Inhibition of COX-1 activity	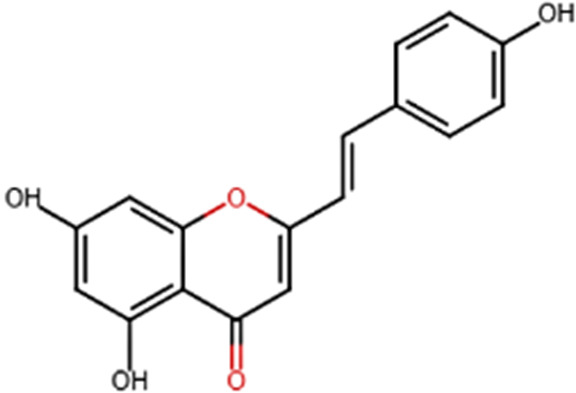
	Inhibition of LTB4 production in human neutrophils	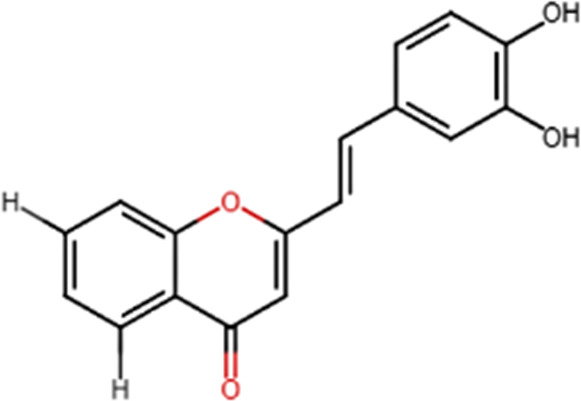

(adapted from Gomes et al., 2010).

## Conclusion and Future Perspectives

Microalgae offer a promising source of various protective and bioactive compounds, which could help protect humans as well as the environment. Cryptophytes are productive in suitable growth conditions, and are biologically active and chemically unique, thus representing secondary metabolites that could be widely used in nutraceuticals, cosmetics and pharmaceuticals. Therefore, they could be used in biomedical applications to maintain or recover human health. The chemical composition of algae is genetically determined, and not all species are capable of producing all compounds. Cryptophytes are fully packed with bioactive compounds; they are extremely rich in ω-3 PUFA, especially in EPA and DHA, as well as in phytosterols. Moreover, they have high-value pigments, i.e. carotenoids and PBPs. They offer, as yet, nearly unexplored source of EPS, vitamins and phenolic compounds with several antioxidant, anti-inflammatory, anti-cancer, anti-Alzheimer’s and other health-promoting effects. Due to their exceptional chemical composition, cryptophytes are already proven to be particularly important food sources in aquatic ecosystems. However, this potential group of algae is nearly untapped in biotechnology. Cryptophytes do not have a recalcitrant cell wall, so compared to many of the already commercially employed algae, they are easier to break and process more for commercial purposes, which also promotes the use of these exceptional algae.

The review highlights the importance of bioactive compounds derived from cryptophyte algae for medical, pharmaceutical, cosmeceutical and food sciences, and it aims to provide new directions for future research. There is little literature associated with cryptophytes, their bioactive components and their functions. In future, further research is needed on the isolation of various bioactive compounds and their efficiency from a growing number of cryptophyte strains. There is also the need to compare the cryptophyte results with information gathered from other algal species. Furthermore, it is essential to determine the optimal growth conditions for the extraction of high quality and sustainable bioactive compounds for commercial use.

## Author Contributions

MA collected sources and wrote the manuscript, designed the figures and Tables. EP and JB contributed to the conception and design of the article and revised it critically for important intellectual content.

## Conflict of Interest

The authors declare that the research was conducted in the absence of any commercial or financial relationships that could be construed as a potential conflict of interest.
